# Evaluation of a two-tier preterm birth prevention service in a tertiary hospital in the United Kingdom: a retrospective cohort study

**DOI:** 10.1186/s12884-025-07538-8

**Published:** 2025-04-15

**Authors:** Michael Shea, Carolina Longo, Valentina LeThanh, Natasja Vandepitte, Joris Hemelaar

**Affiliations:** 1 John Radcliffe Hospital, Oxford University Hospitals NHS Foundation Trust, Oxford, UK; 2 Nuffield Department of Population Health, University of Oxford, Oxford, UK

**Keywords:** Preterm birth, Ultrasound, Cervical length, Progesterone, Cervical cerclage, Risk factor

## Abstract

**Background:**

Preterm birth is the most important cause of neonatal morbidity and mortality. Clinical guidelines recommend assessment of risk of preterm birth and implementation of interventions to reduce preterm birth risk through dedicated preterm birth clinics. We hypothesized that a two-tier preterm birth clinic pathway can safely manage women at the highest risk of preterm birth while reducing intervention for women at moderate risk of preterm birth. We aimed to test this hypothesis by evaluating risk factors, management, and outcomes of women attending a two-tier preterm birth prevention service.

**Methods:**

We conducted a retrospective cohort study of women who gave birth between January and June 2021 at a tertiary hospital in Oxford, UK. We included two cohorts: women attending a Cervical Screening Clinic and women attending a Preterm Birth Clinic, and we also reviewed all cases of births before 34 weeks over that time period. At the initial midwife appointment at 8–10 weeks’ gestation, risk factors for preterm birth were assessed. Pregnant women with moderate risk factors (previous preterm birth at 32^+ 0^ − 33^+ 6^ weeks, previous preterm prelabour rupture of membranes (PPROM) at 32^+ 0^ − 33^+ 6^ weeks, previous LLETZ / cone biopsy, known abnormal uterus, previous caesarean section at 10 cm dilatation, and multiple pregnancy) were referred to the Cervical Screening Clinic for a cervical length scan by a sonographer. Pregnant women with major risk factors (previous preterm birth at 16^+ 0^ − 31^+ 6^ weeks, previous PPROM at less than 32^+ 0^ weeks, radical trachelectomy, previous cervical cerclage) as well as those with a cervix < 25 mm at any scan were referred to the Preterm Birth Clinic for a cervical length scan and counselling by a specialist obstetrician. Detailed information on risk factors, management, and perinatal outcomes were collected from case notes and analysed.

**Results:**

189 women attended the Cervical Screening Clinic: 79.1% had a moderate risk factor for preterm birth, 100% had a cervical length scan, 7% had a short cervix and 4.2% received an intervention. All 196 infants were live born, with overall preterm birth rates of 14.8% at < 37 weeks, 3.1% at < 32 weeks, and 0% at < 28 weeks. The spontaneous live preterm birth rates were 9.7% at < 37 weeks, 2.6% at < 32 weeks and 0% at < 28 weeks. 79 women attended the Preterm Birth Clinic: 87.3% had a major risk factor for preterm birth, 100% had ≥ 1 cervical length scan, 41.3% had a short cervix, 78.1% received vaginal progesterone, and 39% had a cervical cerclage. Overall preterm birth rates were 33.8% at < 37 weeks, 10.3% at < 32 weeks and 4.4% at < 28 weeks. Spontaneous live preterm birth rates were 22.1% at < 37 weeks, 7.4% at < 32 weeks, and 2.9% at < 28 weeks. 115 women gave birth to 130 babies before 34 weeks: 80% had no major risk factor for preterm birth, 29% had a cervical length scan and less than 15% had an intervention. Over 90% had a live birth, but the neonatal death rate was high (8.5%).

**Conclusion:**

Women with moderate risk factors for preterm birth seen in the Cervical Screening Clinic had low rates of intervention and good perinatal outcomes. Most women with major risk factors were appropriately referred and managed by the Preterm Birth Clinic. This two-tier preterm birth prevention service therefore appears safe and effective.

**Supplementary Information:**

The online version contains supplementary material available at 10.1186/s12884-025-07538-8.

## Introduction

Preterm birth (before 37^+ 0^ weeks gestation) is the largest cause of neonatal morbidity and mortality in the UK [1] and prevention of preterm birth is a key element of the Saving Babies’ Lives care bundle [2]. The UK Preterm Clinical Network published guidelines in 2019 emphasizing the importance of assessing the risk of preterm birth and of implementing interventions to reduce that risk [3]. They further highlight the role of dedicated preterm birth clinics, which provide care to asymptomatic women at higher risk of preterm birth. The UK Preterm Clinical Network recommends a triage according to risk factors. Women are considered at high risk of preterm birth if they have had a previous preterm birth or mid-trimester loss (16 to 34 weeks), previous preterm prelabour rupture of membranes (PPROM) at less than 34 weeks, previous cervical cerclage, a known uterine variant, intrauterine adhesions, or history of trachelectomy. They are considered at intermediate risk if they have had a previous caesarean section at full dilatation or a significant cervical excisional event e.g. Large Loop Excision of the Transformation Zone (LLETZ) [3]. Nonetheless, most women who give birth preterm do not have any identifiable risk factors [4]. 

Preterm birth clinics typically offer monitoring via transvaginal cervical length scans and interventions for women with a short cervix (most commonly cervical cerclage or vaginal progesterone) [5]. A short cervical length is strongly associated with preterm birth in asymptomatic women with risk factors for preterm birth, and the shorter the cervix the higher the risk [6]. However, there is less evidence that cervical length scans reduce (rather than simply predict) preterm birth [7]. Cervical cerclage for women with risk factors reduces birth before 34 weeks by about a quarter [8]. Two recent meta-analyses reported that vaginal progesterone reduces preterm birth before 34 weeks by about 20–50% for women with risk factors, and reduces perinatal mortality by a third [9, 10]. The evidence for interventions such as Arabin pessaries or clindamycin is inconclusive [11, 12]. 

There remains, however, significant variation in recommendations from preterm birth related guidelines [13] and in the practices of preterm birth clinics in the UK [14]. Moreover, recent detailed comparative information regarding the performance of preterm birth clinics is lacking. The aim of this study was to assess detailed information on referrals, management, and outcomes of a two-tier preterm birth prevention service consisting of a sonographer-led Cervical Screening Clinic and a doctor-led Preterm Birth Clinic. To better understand the impact of the two-tier preterm prevention service we also reviewed all the women who actually gave birth before 34 weeks’ gestation.

## Methods

### Study design

Two retrospective cohort studies were performed, including women who gave birth at the John Radcliffe Hospital, Oxford, UK, between the 1st of January 2021 and the 30th of June 2021:

Cohort 1: women attending the sonographer-led Cervical Screening Clinic.

Cohort 2: women attending the doctor-led Preterm Birth Clinic.

Initial risk stratification for preterm birth was performed at the first midwife appointment at 8–10 weeks’ gestation, where a standard booking proforma allows assessment of risk factors for preterm birth. Pregnant women with moderate risk factors (previous preterm birth at 32^+ 0^ − 33^+ 6^ weeks, previous PPROM at 32^+ 0^ − 33^+ 6^ weeks, previous LLETZ / cone biopsy, known abnormal uterus, previous caesarean section at 10 cm dilatation, and multiple pregnancy) are referred to the Cervical Screening Clinic for a cervical length scan by a sonographer. Note that the moderate risk factors used in this study overlap with, but are not identical to, the intermediate risk factors defined by the UK Preterm Clinical Network. Pregnant women with major risk factors (previous preterm birth at 16^+ 0^ − 31^+ 6^ weeks, previous PPROM at less than 32^+ 0^ weeks, radical trachelectomy, previous cervical cerclage), as well as women with a cervix < 25 mm at any scan, were referred to the Preterm Birth Clinic for a cervical length scan and counselling by a specialist obstetrician. Pregnant women who were seen in other consultant-led clinics were additionally seen in the Cervical Screening Clinic or Preterm Birth Clinic if they met referral criteria.

We also reviewed the notes of all women who had a preterm birth at less than 34 weeks’ gestation during the same time period. This group of women allowed us to examine what proportion of women who delivered at less than 34 weeks had risk factors for preterm birth and what proportion of these were correctly referred to and managed by the preterm birth service. We report overall preterm birth rates per clinic, which we define as any live birth at less than 37^+ 0^ weeks gestation plus any stillbirth occurring from 24^+ 0^ – 36^+ 6^ weeks. Stillbirth is defined as birth with no signs of life from 24^+ 0^ weeks gestation onwards. We also report spontaneous live preterm birth rates per clinic, defined as any live birth at less than 37^+ 0^ weeks gestation where the onset of labour was spontaneous (regardless of mode of birth). Spontaneous preterm birth excludes births following an induction of labour or a prelabour caesarean section. Mid-trimester miscarriage is defined as birth between 13^+ 0^ and 23^+ 6^ weeks with no signs of life at birth.

### Cervical screening clinic

Women with a moderate risk factor for preterm birth (but no major risk factors) identified at their initial midwife appointment in early pregnancy were referred to the sonographer-led Cervical Screening Clinic at around 20 weeks’ gestation for a single transvaginal ultrasound scan to measure the cervical length. In this clinic, women are seen by a sonographer (not a doctor) and undergo a cervical length scan, but do not have a comprehensive review of their medical history. If the cervical length was normal (≥ 25 mm), the woman was discharged from the clinic. If the cervical length was short (< 25 mm), the woman was referred on to the Preterm Birth Clinic. Sonographers (either radiographer-sonographers or midwife-sonographers) undergo training in transvaginal cervical length monitoring and require accreditation before they can work in the Cervical Screening Clinic. If they identify a short cervix or have concerns about the images they obtained, then the images are reviewed by a fetal medicine consultant from the Preterm Birth team. If a short cervix is confirmed on image review, then the pregnant woman is seen in the next available Preterm Birth Clinic and scanned by a consultant obstetrician. The six moderate risk factors that warranted referral to the Cervical Screening Clinic were: previous preterm birth at 32^+ 0^ − 33^+ 6^ weeks, previous PPROM at 32^+ 0^ − 33^+ 6^ weeks, previous LLETZ / cone biopsy, known abnormal uterus, previous caesarean section at 10 cm dilatation, and multiple pregnancy.

### Preterm birth clinic

Women with a major risk factor for preterm birth identified at their initial midwife appointment in early pregnancy were referred to the consultant-led Preterm Birth Clinic by 16 weeks’ gestation. In this clinic, women are reviewed by a senior obstetrician with a special interest in the management of preterm birth. The five major risk factors that warranted referral to the Preterm Birth Clinic were: previous preterm birth at 16^+ 0^ − 31^+ 6^ weeks, previous PPROM at less than 32^+ 0^ weeks, radical trachelectomy, previous cervical cerclage, and a short cervix (< 25 mm) identified in the Cervical Screening Clinic. The Preterm Birth Clinic offers ongoing monitoring with repeated cervical length scans, and interventions including vaginal progesterone, cervical cerclage (ultrasound or history-indicated) and treatment of vaginal infections. Frequency of monitoring as well as treatment decisions are at the discretion of the responsible consultant.

### Core outcome sets

A core outcome set has been proposed for evaluation of interventions to reduce preterm birth [15]. As this study is a retrospective observational study, we report on the subset of those outcomes for which we have information: gestational age at birth, offspring mortality, and birthweight.

### Data collection, storage, and analysis

Data were extracted from two sources: the electronic patient records (Cerner Millennium Powerchart, Oracle Cerner) and ultrasound reporting software (ViewPoint 6, GE Healthcare). The data included demographic data, risk factors, pregnancy complications, investigations in pregnancy, ultrasound scan data, interventions, and birth outcomes. If patients were transferred from another hospital, data were extracted from the referral letters. Data were held in secure electronic files (password protected Excel files) and immediately anonymised. Data analysis was performed using R version 4.2.1 via R Studio. Where data was incomplete, calculations (e.g. percentages, means, medians) were made using the available data only and the relevant denominators are listed in the tables; no missing data was imputed. Descriptive data is presented as raw numbers and percentages. Statistical tests of significance for categorical data were performed using the Chi square test or Fischer’s exact test (when the sample size was small) in R Studio using R version 4.2.1.

## Results

### Women attending the cervical screening clinic

**Fig. 1:**
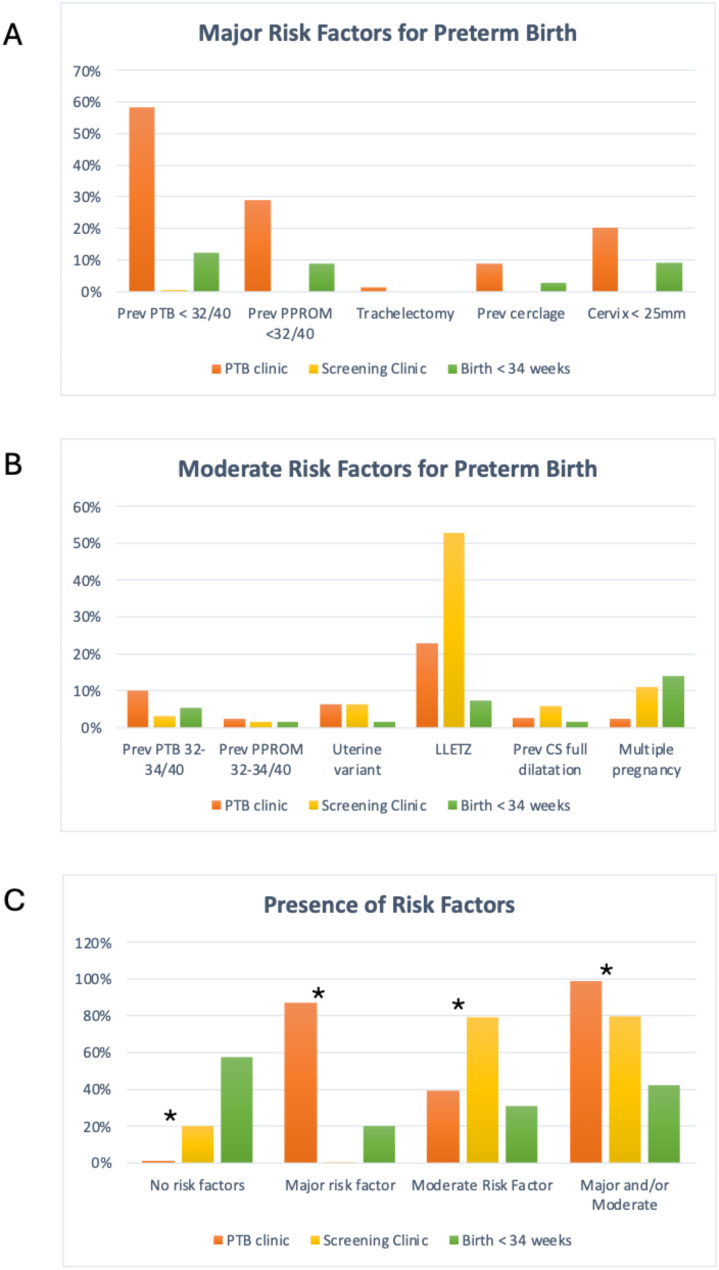
Risk factors for preterm birth. Percentage of women from the different cohorts having major risk factors (**A**), moderate risk factors (**B**) or a combination of risk factors (**C**). Risk factors are not mutually exclusive. The major risk factor of a short cervix (< 25 mm) refers to the time of the first clinic visit for women who attended the Preterm Birth Clinic or the Screening Clinic. Asterix (*) indicates a significant difference between the Preterm Birth Clinic and Screening Clinic with *p* < 0.01. Abbreviations: 32/40 = 32 weeks’ gestation, CS = caesarean section, LLETZ = Large Loop Excision of the Transformation Zone, PPROM = preterm prelabour rupture of membranes, Prev = previous, PTB = preterm birth

189 women attended the Cervical Screening Clinic (Table 1). Most women (79.1%) had at least one moderate risk factor, most commonly a history of a LLETZ procedure (52.9%). Additionally, 1 (0.5%) woman had a major risk factor (Table 1; Fig. 1).

**Table 1:** Demographic characteristics and risk factors for preterm birth

	Cervical Screening Clinic	Preterm Birth Clinic	Birth prior to 34 weeks
n =	189 women, 196 fetuses	79 women, 82 fetuses	115 women, 130 fetuses
**Demographics**			
• Age at delivery	Mean 32.8, SD 4.4	Mean 32.0, SD 4.9	Mean 31.2, SD 6.3
• Parity	Median 1, range 0–4 P0: 67/188 (35.6%) *P* ≥ 1: 121/188 (64.4%)	Median 1, range 1–6 P0: 13/79 (16.5%) *P* ≥ 1: 66/79 (83.5%)	Median 0, range 0–4 P0: 60/113 (53.1%) *P* ≥ 1: 53/113 (46.9%)
• BMI	Mean 25.6, SD 5.0	Mean 26.9, SD 6.0	Mean 26.8, SD 6.8
• Ethnicity	Asian: 9/189 (4.8%) Black: 3/189 (1.6%) Mixed: 25/189 (5.3%) White British: 128/189 (67.7%) White Other: 24/189 (12.7%)	Asian: 5/56 (8.9%) Black: 2/56 (3.6%) Mixed: 2/56 (3.6%) White British: 41/56 (73.2%) White Other: 6/56 (10.7%)	Asian: 17/115 (14.8%) Black: 6/115 (5.2%) Mixed: 16/115 (13.9%) White British: 62/115 (53.9%) White other: 14/115 (12.1%)
**Pregnancy complications in index pregnancy**			
• Hypertensive disorders	NR	4 / 64 (6.3%)	18 / 114 (15.8%)
• GDM	NR	9 / 64 (14.1%)	9 / 114 (7.9%)
• Significant infection	NR	4 / 64 (6.3%)	21 / 114 (18.4%)
• COVID-19	NR	5 / 65 (7.7%)	9 / 114 (7.9%)
• APH	NR	13 / 66 (19.7%)	15 / 114 (13.2%)
• Praevia / accreta	NR	3 / 71 (4.3%)	8 / 114 (7.0%)
• OC / ICP	NR	0 / 64 (0%)	0 / 114 (0%)
• SGA on scan	NR	2 / 65 (3.1%)	32 / 129 (24.8%)
• Fetal anomaly on scan	NR	3 / 64 (4.7%)	10 / 129 (7.8%)
• Any complication	NR	31 / 64 (48.4%)	76 / 110 (69.1%)
**Major Risk Factors for PTB**			
• Prev PTB or loss 16/40–31^+ 6^/40	1 / 187 (0.5%)	46 / 79 (58.2%)	14 / 113 (12.4%)
• Prev PPROM < 32/40	0 / 187 (0%)	23 / 79 (29.1%)	10 / 113 (8.8%)
• Trachelectomy	0 / 187 (0%)	1 / 79 (1.3%)	0 / 108 (0%)
• Prev cerclage	0 / 187 (0%)	7 / 79 (8.9%)	3 / 112 (2.7%)
• Short cervix *	NA	16 / 79 (20.3%)	10 / 107 (9.3%)
Total Major Risk factors for PTB	0 risk: 186 / 187 (99.5%) 1 risk: 1 / 187 (0.5%)	0 risk: 10 / 79 (12.7%) 1 risk: 46 / 79 (58.2%) 2 risks: 22 / 79 (27.8%) ≥ 3 risks: 1 / 79 (1.3%)	0 risk: 88 / 110 (80.0%) 1 risk: 11 / 110 (10%) 2 risks: 9 / 110 (8.2%) ≥ 3 risks: 2 / 110 (1.8%)
Proportion of women with ≥ 1 major risk factor	1 / 187 (0.5%)	69 / 79 (87.3%)	For PTB < 34 weeks’: 22 / 110 (20%) For spont PTB < 34 weeks’: 8 / 47 (17%) For PTB at: < 28 weeks’: 9 / 37 (24.3%) 28–31^+ 6^ weeks’: 7 / 38 (18.4%) 32–33^+ 6^ weeks: 6 / 35 (17.1%)
**Moderate Risk factors for PTB**			
• Prev PTB 32^+ 0^ – 33^+ 6^	6 / 188 (3.2%)	8 / 79 (10.1%)	6 / 113 (5.3%)
• Prev PPROM 32^+ 0^ – 33^+ 6^	3 / 187 (1.6%)	2 / 79 (2.5%)	2 / 113 (1.8%)
• Known uterine variant	12 / 187 (6.4%)	5 / 79 (6.4%)	2 / 110 (1.8%)
• LLETZ / cone biopsy	99 / 187 (52.9%)	18 / 79 (22.8%)	8 / 110 (7.3%)
• Prev CS at full dilatation	11 / 187 (5.9%)	2 / 76 (2.6%)	2 / 110 (1.8%)
• Multiple pregnancy	21 / 189 (11.1%)	2 / 79 (2.5%)	16 / 114 (14%)
Proportion with ≥ 1 moderate risk factor	148 / 187 (79.1%)	30 / 76 (39.4%)	For PTB < 34 weeks: 33 / 106 (31.1%) For spont PTB < 34 weeks: 18 / 47 (38.3%)
Proportion with at least one moderate or major risk factor	149 / 187 (79.7%)	75 / 76 (98.7%)	45 / 106 (42.5%)
**Minor Risk Factors for PTB**			
• Smoking	16 / 188 (8.5%)	15 / 71 (21.1%)	12 / 113 (10.6%)
• Age < 18	0 / 188 (0%)	0 / 79 (0%)	1 / 115 (0.9%)
• Age > 40	6 / 188 (3.2%)	2 / 79 (2.5%)	9 / 115 (7.8%)
• BMI < 18.5	4 / 187 (2.1%)	2 / 70 (2.9%)	7 / 111 (6.3%)
• UTI in pregnancy	NR	12 / 69 (17.4%)	NR

* short cervix: < 25 mm on transvaginal ultrasound scan

† SGA on scan: estimated fetal weight < 10^th^ centile

Abbreviations: APH = Antepartum haemorrhage, BMI = Body Mass Index, CS = Caesarean Section, GDM = Gestational Diabetes Mellitus, OC / ICP = Obstetric Cholestasis / Intrahepatic Cholestasis of Pregnancy, LLETZ = Large Loop Excision of the Transformation Zone, NA = Not Applicable, NR = Not Recorded, SD = Standard Deviation, PTB = Preterm Birth, PPROM = Preterm Prelabour Rupture of Membranes, SGA = Small for Gestational Age, UTI = Urinary Tract Infection

All the women seen in the Cervical Screening Clinic had a transvaginal ultrasound scan for cervical length (Table 2). A short cervix (< 25 mm) was identified on 7% of scans (Table 2). Of the 13 women found to have a short cervix in the Cervical Screening Clinic, 10 were referred to the Preterm Birth Clinic and 3 were discussed with or referred to the fetal medicine team.

**Table 2:** Monitoring for risk of preterm birth

	Cervical Screening Clinic	Preterm Birth Clinic	Birth prior to 34 weeks
n =	187 women	79 women	107 women
**TVS for cervical length**			
• Number of scans	≥ 1 scan: 187 / 187 (100%)	≥ 1 scan: 79 / 79 (100%) 1 scan: 19 / 79 (24.1%) 2 scans: 27 / 79 (34.2%) 3 scans: 18 / 79 (22.8%) 4 scans: 10 / 79 (12.7%) ≥ 5 scans: 5 / 79 (6.3%)	≥ 1 scan: 31 / 107 (29.0%) 1 scan: 14 / 107 (13.1%) 2 scans: 9 / 107 (8.4%) 3 scans: 5 / 107 (4.7%) 4 scans: 2 / 107 (1.9%) ≥ 5 scans: 1 / 107 (0.9%)
• Gestation at first TVS	Median: 20.4 weeks Range: 13–26 weeks	Median: 16 weeks Range: 10–26 weeks	Median: 19 weeks Range: 11–22 weeks
• Interscan intervals	NR	≤ 1 week: 17 / 113 (15.0%) 2 weeks: 30 / 113 (26.5%) 3 weeks: 26 / 113 (23.0%) 4 weeks: 21 / 113 (18.6%) ≥ 5 weeks: 19 / 113 (16.8%)	NR
• Short cervix (< 25 mm) identified	On at least one scan: 13 / 187 (7.0%)	On 1^st^ scan: 18 / 79 On 2^nd^ scan: 18 / 60 On 3^rd^ scan: 6 / 33 On 4^th^ scan: 4 / 15 On 5^th^ scan: 0 / 5 On ≥ 1 scan: 31/75 (41.3%)	On 1^st^ scan: 8 / 31 On 2^nd^ scan: 6 / 17 On 3^rd^ scan: 2 / 8 On 4^th^ scan: 1 / 3 On 5^th^ scan: 1 / 1 On ≥ 1 scan: 10/31 (32.3%)
**Vaginal swabs**			
• Swab sent	NR	71 / 79 (89.9%)	NR
• Swab result	NR	Negative: 42 / 71 (59.2%) Positive: 29 / 71 (40.8%) Candida: 16 / 71 (22.5%) Mixed anaerobes: 14 / 71 (19.7%) Group B strep: 4 / 71 (5.6%) *S. ludgunensis*: 2 / 71 (2.8%) *Staph aureus*: 1 / 71 (1.4%)	NR
**STI screening**			
• STI screen performed	NR	63 / 79 (79.7%)	NR
• STI screen result	NR	Negative: 62 / 63 (98.4%) Trichomonas: 1 / 63 (1.6%)	NR
Urine MSU			
• MSU sent	NR	78 / 79 (98.7%)	NR
• MSU result	NR	Negative: 47 / 78 (61.5%) Mixed growth: 21 / 78 (26.9%) *E. coli*: 6 / 78 (7.7%) *E. faecalis*: 3 / 78 (3.8%) Group B strep: 1 / 78 (1.3%)	NR

Abbreviations: MSU = Mid-Stream Urine, STI = Sexually Transmitted Infection

**Fig. 2:**
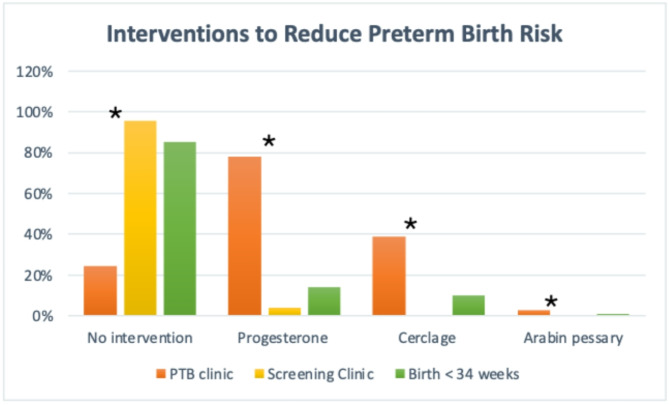
Interventions to reduce preterm birth risk. Interventions to reduce the risk of preterm birth across the three cohorts. The interventions listed (vaginal progesterone, cervical cerclage, and Arabin pessary) are not mutually exclusive. Asterix (*) indicates a significant difference between the Preterm Birth Clinic and Screening Clinic with *p* < 0.01

Only eight women from the Cervical Screening Clinic (4.2%) received vaginal progesterone. Seven of these had a short cervix on scan (< 25 mm) and one woman had a borderline cervical length (26 mm). None of the women from the Cervical Screening Clinic had a cervical cerclage or Arabin pessary (Table 3; Fig. 2). Most women (95.8%) seen in the Cervical Screening Clinic had no interventions to reduce the risk of preterm birth.

**Table 3:** Interventions to reduce preterm birth

	Cervical Screening Clinic	Preterm Birth Clinic	Birth prior to 34 weeks
n =	189 women	79 women	115 women
PV/PR progesterone			
• Progesterone prescribed	8 / 187 (4.2%)	57 / 73 (78.1%)	15 / 108 (13.9%)
• Gestation started	Median: 20.16 weeks Range: 19–23 weeks	Median: 15.5 weeks Range: 12–25 weeks	Median: 17 weeks Range: 12–22 weeks
Cervical cerclage			
• Cerclage inserted	0 / 182 (0%)	30 / 77 (39.0%)	11 / 113 (9.7%)
• Indication	NA	History: 7 / 26 (26.9%) US: 13 / 26 (50%) Emergency: 6 / 26 (23.1%)	History: 3 / 10 (30%) US: 7 / 10 (70%)
• Type of cerclage	NA	Vaginal: 28 / 30 (93.3%) Abdominal 2 / 30 (6.7%)	Vaginal: 7 / 9 (77.8%) Abdominal: 2 / 9 (22.2%)
• Gestation	NA	Median: 20 weeks Range: 12–25 weeks (+ 1 preconception)	Median: 21 weeks Range 13–24 weeks (+ 2 preconception)
Arabin pessary			
• Arabin pessary	0 / 182 (0%)	2 / 72 (2.7%)	1 / 113 (0.9%)
• Gestation	NA	18 weeks: 2 / 2	23 weeks: 1 / 1
Combinations of interventions	NA	None: 19 / 78 (24.3%) Prog only: 28 /78 (35.9%) Cerc only: 1 / 78 (1.3%) Prog + Cerc: 28 / 78 (35.9%) Arabin only: 1 / 78 (1.3%) Prog + Arabin: 1 / 78 (1.3%)	None: 92 / 108 (85.2%) Prog only: 6 / 108 (5.6%) Cerc only: 1 / 108 (0.9%) Prog + Cerc: 8 / 108 (7.4%) Prog + Cerc + Arabin: 1/108 (0.9%)
Clindamycin prescribed	NR	26 / 79 (32.9%)	NR

Abbreviations: Arabin = arabin pessary, Cerc = Cervical cerclage, Prog = progesterone, NA = Not Applicable, NR = Not Recorded, PV/PR = per vagina / per rectum, US = ultrasound

The 189 women who were seen in the Cervical Screening Clinic gave birth to 196 infants for which we have birth data (Table 4). All the births were livebirths. The preterm birth rate was higher than in the general population, with 14.8% of women giving birth before 37 weeks, 3.1% giving birth before 32 weeks, but none before 28 weeks. The spontaneous live preterm birth rates were 9.7% at < 37 weeks, 2.6% at < 32 weeks and 0% at < 28 weeks.

**Table 4:** Birth outcomes

		Cervical Screening Clinic		Preterm Birth Clinic		Birth prior to 34 weeks	
n =		181 women, 196 fetuses		79 women, 82 fetuses		115 women, 130 fetuses	
**Gestation at birth**							
Gestation of live births		Median = 39^+ 2^ Range = 29^+ 2^ – 42^+ 4^		Median = 38^+ 4^ Range: 23^+ 2^ – 41^+ 4^		Median: 29^+ 5^ Range: 20–33^+ 6^ < 28 weeks’: 41 / 118 = 34.7% 28^+ 0^ – 31^+ 6^ weeks: 41 / 118 = 34.7% 32–33^+ 6^ weeks’: 36 / 118 = 30.5%	
Live birth ≥ 37/40		167 / 196 (85.2%)		45 / 68 (66.2%)		NA	
Live birth < 37/40		29 / 196 (14.8%)		23 / 68 (33.8%)		NA	
Spont live birth < 37/40		19 / 195 (9.7%)		15 / 68 (22.1%)		NA	
Live birth < 34/40		6 / 196 (3.1%)		11 / 68 (16.2%)		NA	
Spont live birth < 34/40		5 / 195 (2.6%)		6 / 68 (8.9%)		NA	
Live birth < 32/40		6 / 196 (3.1%)		7 / 68 (10.3%)		NA	
Spont live birth < 32/40		5 / 195 (2.6%)		5 / 68 (7.4%)		NA	
Live birth < 28/40		0 / 196 (0%)		2 / 68 (2.9%)		NA	
Spont live birth < 28/40		0 / 195 (0%)		2 / 68 (2.9%)		NA	
**Birth outcomes**							
Live birth		195 / 195 (100%)		68 / 73 (93.2%)		118 / 130 (90.8%)	
Mid-trimester miscarriage		NR		3 / 73 (4.1%) Gestation: 14^+ 1^ – 22^+ 0^		NR	
Stillbirth		0 / 195 (0%)		2 / 73 (2.7%)		12 / 130 (9.2%) < 28 weeks’: 7 / 48 (14.6%) 28^+ 0^ – 31^+ 6^ weeks’: 2 / 43 (4.7%) 32–33^+ 6^ weeks’: 3 / 39 (7.7%)	
Neonatal deaths (% of livebirths)		0 / 195 (0%)		0 / 73 (0%)		10 / 118 (8.5%) < 28 weeks’: 8 / 41 (19.5%) 28^+ 0^ – 31^+ 6^ weeks’: 2 / 41 (4.9%) 32–33^+ 6^ weeks’: 2 / 36 (5.6%)	
Alive at 28 days from total births:		195 / 195 (100%)		68 / 73 (93.2%)		108 / 130 (83.1%) < 28 weeks’: 33 / 48 (68.8%) 28^+ 0^ – 31^+ 6^ weeks’: 40 / 43 (93.0%) 32–33^+ 6^ weeks’: 35 / 39 (89.7%)	
**Birth details**							
Initiation of labour / birth		Spont: 97 / 195 (49.7%) Initiated: 98 / 195 (50.3%)		Spont: 36 / 69 (52.1%) Initiated: 33 / 69 (47.8%)		See below	
Initiation of labour / birth for PTB < 37/40		Spont: 19 / 29 (65.6%) Initiated: 10 / 29 (34.5%)		Spont: 16 / 24 (66.7%) Initiated: 8 / 24 (33.3%)		See below	
Initiation of labour / birth for PTB < 34/40		Spont: 5 / 6 (83.3%) Initiated: 1 / 6 (16.6%)		Spont: 7 / 11 (63.6%) Initiated: 4 / 11 (36.4%)		Spont 47 / 130 (36.2%) Initiated 83 / 130 (63.8%)	
Mode of birth		SVD: 86 / 195 (44.1%) Instrum: 35 / 195 (17.4%) CS: 75 / 195 (38.5%)		SVD: 30 / 63 (47.6%) Instrum: 10 /63 (15.9%) CS: 23 / 63 (36.5%)		SVD: 52 / 130 (40%) Instrum: 0 / 130 (0%) CS: 78 / 130 (60%)	
Birthweight		Median: 3290 g Range: 900–5910 g		Median: 2962.5 g Range: 490–4775 g		Median:1163 g Range: 270–2520 g	
SGA at birth < 10^th^ centile		11 / 194 (5.7%)		8 / 62 (12.9%)		37 / 124 (29.8%)	
SGA at birth < 3^rd^ centile		1 / 194 (0.5%)		3 / 62 (4.8%)		24 / 124 (19.4%)	
Aetiology of PTB (primary cause documented for PTB)		NR		NR		Chorio: 37 / 130 (28.5%) IUGR: 15 / 130 (11.5%) Fetal Distress: 14 / 130 (10.8%) PET/eclampsia: 12 / 130 (9.2%) Multifetal gest.: 12 / 130 (9.2%) None identified: 11 / 130 (8.4%) APH(late)/abrupt.: 10 / 130 (7.7%) IUD: 5 / 130 (3.8%) Fetal anomaly: 4 / 130 (3.1%) Sepsis: 3 / 130 (2.3%) Maternal condition: 2 / 130 (1.5%) Plac. praevia/PAS: 2 / 130 (1.5%) APH (early): 1 / 130 (0.8%) Cord prolapse: 1 / 130 (0.8%)	

Abbreviations: 28d = 28 days, abrupt = abruption, APH = antepartum haemorrhage, Chorio = chorioamnionitis, ELCS = Elective caesarean section, EMCS = emergency caesarean section, gest = gestation, Instrum = instrumental birth (forceps or ventouse), IUD = intrauterine death, IUGR = intrauterine growth restriction, NA = Not Applicable, ND = neonatal death, NR = Not Recorded, PAS = placenta accrete spectrum, PET = preeclampsia, plac. = placenta, Spont = spontaneous, SVD = spontaneous vaginal delivery

### Women attending the preterm birth clinic

79 women attended the Preterm Birth Clinic (Table 1). 87.3% had at least one major risk factor for preterm birth. The most common risk factor was previous preterm birth or mid-trimester loss between 16^+ 0^ and 31^+ 6^ weeks’ gestation (58.2%) (Table 1; Fig. 1). Additionally, 38.1% of the women had at least one moderate risk factor, and overall 98.7% of women had at least one major or moderate risk factor (Table 1; Fig. 1).

Every woman seen in the Preterm Birth Clinic had at least one cervical length measurement and most women had serial cervical length scans (Table 2). Overall, 41.3% of the women seen in the preterm birth clinic had a short cervix (< 25 mm) identified on at least one scan (Table 2).

Most women seen in the Preterm Birth Clinic had screening for genital infection; low vaginal swab culture was positive in 40.8% of cases (Table 2). The screening for sexually transmitted infection was negative in 98.4% of cases, with just one positive result (Table 2). Urine culture was almost universal, with 98.7% having at least one midstream urine sample sent. Urine culture was positive (single organism isolated, not mixed growth) on at least one occasion for 17.4% of the women (Table 2).

The most common intervention to reduce the risk of preterm birth was progesterone pessary (78.1% of women) (Table 3; Fig. 2). Progesterone pessaries may be taken vaginally or rectally; intramuscular progesterone injections are not used in our Preterm Birth Clinic. The median gestation for starting progesterone was 15.5 weeks (range of 12–25 weeks). Cervical cerclage was the second most common intervention (39%) (Table 3; Fig. 2). Most of the cerclages (93%) were placed vaginally. Two women had transabdominal cerclages. Using the definitions from the RCOG green-top guidance on cervical cerclage [16], these cerclages can be classified as ultrasound-indicated in half the cases (50%), history-indicated in roughly a quarter (26.9%), and emergency cerclage in the remaining quarter (23.1%) (Table 3). Eligibility for history-indicated cerclage and number of cerclages placed is explored in more detail in Table S3. The median gestation for cerclage was 20 weeks (range 12–25 weeks and one transabdominally pre-pregnancy). Arabin pessaries were used infrequently (2.7%). About a third of the women (32.9%) were treated with clindamycin with the aim of reducing the risk of preterm birth. Clindamycin treatment was given either in response to a positive swab or in some cases based on symptoms suggestive of bacterial vaginosis or based on a history of previous preterm birth in the context of genital tract infection (Table 3; Fig. 2).

Pregnancy outcome was available for 73 out of 79 women attending the Preterm Birth Clinic, most of whom (93.2%) had a live birth (Table 4). Three women (4.1%) had a mid-trimester miscarriage (after having been seen in the Preterm Birth Clinic), and two women (2.7%) had a stillbirth. There were no neonatal deaths (Table 4). Of the women who had a live birth, 33.8% gave birth before 37 weeks, 16.2% before 34 weeks, 10.3% before 32 weeks and 4.4% before 28 weeks. The spontaneous live preterm birth rates were 22.1% at < 37 weeks, 7.4% at < 32 weeks, and 2.9% at < 28 weeks. 12.9% of neonates were below the 10^th^ centile of birthweight for gestational age and 4.8% below the 3^rd^ centile using the Fetal Medicine Foundation birthweight chart [17] (Table 4). More detailed outcomes are presented in Table S2 for women who attended the Preterm Birth Clinic and who delivered prior to 34 weeks gestation.

### Women who gave birth at less than 34 weeks’ gestation

115 women gave birth to 130 neonates at less than 34 weeks’ gestation (Table 1). 36% of these births had a spontaneous onset, and 64% were caregiver initiated (Table 4). Within the caregiver-initiated group, several cases were documented to have PPROM and then went on to have an induction of labour or pre-labour caesarean section. PPROM is often a precursor to spontaneous preterm birth. However, induction of labour or pre-labour caesarean before 34^+0^ weeks gestation would only be offered in response to a complication in pregnancy (likely but not definitely related to the PPROM). These cases therefore fall into a grey zone between clearly spontaneous and clearly iatrogenic preterm birth. Most women, 88/110 (80%), had no major risk factors for preterm birth, and most women, 73/106 (68.9%), had no moderate risk factors for preterm birth. The prevalence of risk factors was similar when considering only spontaneous labours prior to 34 weeks. Overall, 61/106 (57.5%) had neither a major nor a moderate risk factor for preterm birth (Table 1; Fig. 1).

22 women (20%) had a major risk factor for preterm birth, and for 19 we had adequate antenatal information (one was a concealed pregnancy, two were late transfers from another hospital). 15 of the 19 (78.9%) were managed according to Preterm Birth Clinic protocols, one underwent a cervical length scan only in the referring hospital, and three women had no assessment for risk of preterm birth. Furthermore, 33/106 (31.1%) who gave birth before 34 weeks had moderate risk factors. Of these, 21 were appropriately seen and had a cervical length scan, but 12/33 (36%) were not (Table 1).

31/107 (29%) of the women who gave birth before 34 weeks’ gestation had a cervical length scan (Table 2) and about half of these women had serial scans. Of the 31 women who had a cervical length scan, 10 (32.3%) had a short cervix identified (i.e., 8.6% of the entire cohort of 115 women) (Table 2).

93/108 (85.2%) of the women who gave birth before 34 weeks’ gestation did not receive any intervention to reduce the risk of preterm birth (Table 3; Fig. 2). Progesterone was prescribed for 15/108 (13.9%), 11/113 (9.7%) had a cerclage placed, and less than 1% had an Arabin pessary. 16 women (14.8%) had at least one intervention (Table 3; Fig. 2).

Over 90% of women who gave birth before 34 weeks gestation had a live birth, but the neonatal death rate was high (8.5%) (Table 4). Babies born before 28 weeks had worse outcomes, with only 33/48 (68.8%) alive at 28 days, due to a combination of a high stillbirth rate (14.6%) and a high neonatal death rate (19.5%) (Table 4). In contrast, for babies born between 28 to 31^+6^ weeks, 40/43 (93.0%) were alive at 28 days. The most common causes of preterm birth were chorioamnionitis (28.5%), intrauterine growth restriction (IUGR) (11.5%), fetal distress (10.8%), pre-eclampsia/eclampsia (9.2%), and multifetal gestation (9.2%) (Table 4). Among the births before 34 weeks, 29.8% were below the 10^th^ centile and 19.4% below the 3^rd^ centile for gestational age.

### Comparison of the two cohorts and the cases with birth at less than 34 weeks

Risk factors, monitoring, and interventions for the Cervical Screening Clinic cohort and the Preterm Birth Clinic cohort can be compared across Tables 1, 2 and 3. Preterm birth rates below 37 weeks for the general population, the Cervical Screening Clinic, and the Preterm Birth Clinic are 8%,^3^ 14.8%, and 33.8% respectively. Similarly, the preterm birth rates below 32 weeks for the general population, the Cervical Screening Clinic, and the Preterm Birth Clinic are 1.4%,^3^ 3.1%, and 10.3% respectively. The pregnancy loss rate from the Preterm Birth Clinic was 6.8%. All women attending the Cervical Screening Clinic had live births, and none gave birth before 29 weeks’ gestation. Over 90% of women who gave birth before 34 weeks’ gestation had a live birth, but the neonatal death rate was high (8.5%).

## Discussion

Two-tier preterm birth services are used to manage the relatively large number of pregnancies at increased risk of preterm birth, in the context of limited consultant obstetrician clinic capacity. However, the populations managed in these two-tier preterm birth services have not been well described in the literature. To the best of our knowledge, this is the first detailed description of a two-tier preterm birth prevention service, in which women at moderate risk of preterm birth were managed in a sonographer-led Cervical Screening Clinic and women at high risk in a doctor-led Preterm Birth Clinic. Women managed via the Cervical Screening Clinic had low rates of intervention and good perinatal outcomes, and women managed via the Preterm Birth Clinic had outcomes in line with their significant risk factors.

Most referrals to the preterm birth prevention service were appropriate. The group of births at less than 34 weeks allowed us to assess a subset of missed referrals to the preterm birth prevention service, as we can see what proportion of women with risk factors who went on to have a preterm birth were not seen in the preterm birth prevention service. About 1 in 4 women with risk factors who gave birth before 34 weeks did not follow the preterm birth referral pathway. Moreover, while the women seen in the Preterm Birth Clinic overall had significantly greater risk factors than those seen in the Cervical Screening Clinic, the referral process was not perfect, as seen from the fact that 12.7% of women in the Preterm Birth Clinic did not have a major risk factor, and 20.9% of women in the Cervical Screening Clinic did not have a moderate risk factor. Improved education and automated referral pathways may help reduce missed referrals and over-referrals. However, the majority of women who gave birth before 34 weeks’ gestation had no major risk factors for preterm birth. Even with a perfect referral system, the Preterm Birth Clinic would see less than a quarter of the women who go on to give birth at less than 34 weeks’. General early pregnancy care to reduce the risk of preterm birth (for example smoking cessation, and treating genital or urinary tract infections) therefore remains essential.

A strength of this study is that it includes three complementary ‘preterm birth’ groups: women with moderate risk factors, women with major risk factors, and women who actually gave birth before 34 weeks (most of whom had no major or moderate risk factors). A second strength of this study is the level of detail provided with regards to referral, management, and outcomes in a large tertiary centre in the UK, which will be useful for those developing or assessing preterm birth clinics in other settings.

This study is limited by the retrospective nature of the data and possible incomplete recording in the electronic medical records. For example, the presence of risk factors is established by the midwife using a standardised questionnaire that may miss some risks (such as PPROM prior to 34 weeks if the woman then gives birth after 34 weeks). Some women seen in the Preterm Birth Clinic were referred from other hospitals. These cases had limited demographic or outcome data.

Large studies of universal cervical screening have found that approximately 2% of low-risk women have a short cervix (< 25 mm) at 18–23 weeks [18, 19]. In contrast, roughly a third of women with a previous preterm birth will have a short cervix in subsequent pregnancies [10]. In our study, less than a third of the women who gave birth before 34 weeks had a cervical length scan, of whom 29% had a short cervix identified. Looked at from a different perspective, 71% of the women who gave birth before 34 weeks and who had a transvaginal ultrasound of the cervix had a normal cervical length. Universal cervical length screening is associated with decreases in hospital admission for threatened preterm labour [20] and in preterm birth [21]. However, the decrease in preterm birth at less than 34 weeks’ was small (from 1.9 to 1.7%), given the costs of universal screening [21]. Moreover, some interventions, such as cervical cerclage, have not been shown to benefit low-risk women with a short cervix [16], and the UK National Screening Committee advises against routine cervical length screening [22]. While some countries (such as Israel) have implemented universal cervical length screening, it is not practiced in most countries. In a recent survey of attendees at the European Spontaneous Preterm Birth Congress, the majority of respondents from a range of countries did not carry out universal cervical length screening, but only targeted screening for women with risk factors for preterm birth [23]. Due to the resources needed, universal cervical screening is not achievable in the short-term for most hospitals in most countries. A two-tier approach to preterm birth prevention is a more cost-effective alternative, as a Cervical Screening Clinic for women at moderate risk of preterm birth can be run by a sonographer alone and only the highest risk women are seen in the doctor-led Preterm Birth Clinic. Our study shows that this approach is safe and effective and may be applied in other settings. Prediction of preterm birth might be improved in the future by integrating tools such as universal cervical length screening, fetal fibronectin testing [24], new biomarkers, or better predictive algorithms (including harnessing artificial intelligence technology). Integrated prediction models such as the QUIPP app are already taking steps in this direction [25].

The effectiveness of the two-tier preterm birth prevention service can only be assessed indirectly as there is no direct control group of pregnant women who would have been eligible for this level of care but who were not offered it. We can therefore compare the outcomes from our Preterm Birth Clinic to outcomes from preterm birth clinics in different settings or we can extrapolate the effects of the interventions offered in our clinic from published standards. Overall, the pregnancy loss (6.8%) and the preterm birth (33.8%) rates were higher for women seen in the Preterm Birth Clinic than the rates in the general population. However, these women have significant obstetric risk factors, and their outcomes are similar to those of women seen in other preterm birth clinics around the world. In a systematic review of preterm birth clinics [5], the preterm birth rates ranged from 15%^24^ to 50%^25^, and pregnancy loss rates from 0.9%^26^ to 8.7%^27^. We find similar results in our updated search of the preterm birth clinic literature (Table S1). Unfortunately, none of the publications that we identified provided separate data for a screening and preterm birth clinic. This limits our ability to compare our results to those of other units, but also highlights the gap in the literature in describing a two-tier preterm birth clinic approach.

We can also extrapolate the overall effect of our Preterm Birth Clinic based on the effectiveness of the interventions offered in that clinic. There is good evidence for the benefits and harms of interventions such as cervical cerclage and progesterone pessaries in an appropriately selected population. In women with risk factors for preterm birth and with a short cervix on ultrasound scan, cervical cerclage reduces preterm birth before 34 weeks with a RR 0.77 (95% confidence interval 0.66 to 0.89) [8]. In that same population, vaginal progesterone reduces preterm birth before 34 weeks with an odds ratio of 0.50 (95% credible interval 0.34 to 0.70) [10]. In our Preterm Birth Clinic, 58 women (73% of the cohort) with risk factors for preterm birth had a cervical cerclage and/or vaginal progesterone, including all (100%) of the 31 women with a short cervix identified on scan.

The observed preterm birth rate at less than 34 weeks in our study was 16% (both in the whole Preterm Birth Clinic cohort and in the subset of women who received an intervention). As all the women with risk factors for preterm birth and a short cervix had appropriate treatment, we can extrapolate that this preterm birth rate would have been higher without treatment. For a relative risk reduction of 25–50% (based on the systematic reviews quoted above), we anticipate that the untreated preterm birth rate in our cohort would have been 21.3–32%. The extrapolated effect of our Preterm Birth Clinic is therefore an absolute risk reduction of 5.3–16% for preterm birth at less than 34 weeks, equating to a number needed to treat of 6 to 19 to prevent one birth at less than 34 weeks. We acknowledge however that these numbers are extrapolated, not measured, apply only to the highest risk cohort of pregnant women, and should be taken cautiously as an illustration of the likely effect of the Preterm Birth Clinic, not its true measured effect.

A second point when considering the outcomes of our Preterm Birth Clinic pathway is the harm from intervention. This is particularly relevant for surgical interventions like a cervical cerclage (see Table S3). A key aspect of our pathway is the use of a Screening Clinic to reduce the burden of intervention on women who have risk factors for preterm birth but are not at the very highest risk of preterm birth. Of the 189 women assessed in our Screening Clinic, only 4% received vaginal progesterone, none underwent a cervical cerclage, and the preterm birth rate at less than 34 weeks was only 3%, with no extremely preterm births. In a perfect service, these women would also have had low intervention rates had they been seen in the Preterm Birth Clinic. However, data from obstetrics more widely shows that the location of care and the type of care provider are correlated with intervention levels. For example, NICE guidance on Intrapartum Care points out that low-risk multiparous women who give birth in Midwife-led units have lower intervention rates in labour then low-risk multiparous women who give birth in Consultant-led units. Our study therefore shows not only the benefits of intervention through the Preterm Birth Clinic, but also the safety of reducing interventions in a group of women at moderate risk of preterm birth.

A key feature of our set-up is the early triage to either a Cervical Screening or Preterm Birth Clinic. The UK Preterm Birth Guidelines do not recommend a separate cervical screening clinic, but they do recommend a two-tier follow-up system: a preterm birth clinic by 12 weeks for all, but the frequency of cervical length scans then depends on risk factors. To the best of our knowledge, all preterm birth clinics described in the literature have a single-tier model where all patients are assessed in a single preterm birth clinic, regardless of whether they are at high or intermediate risk [26–37]. Our triage system, with a Cervical Screening Clinic that is sonographer-led, appears to be safe (with no births under 29 weeks, and all live births), and avoids having a Preterm Birth Clinic appointment for the vast majority of women with intermediate risk factors.

## Conclusion

Women with moderate risk factors for preterm birth seen in the Cervical Screening Clinic had low rates of intervention and good perinatal outcomes. Most women with major risk factors for preterm birth were appropriately referred and managed by the Preterm Birth Clinic. Most women who delivered < 34 weeks did not have any major risk factors for preterm birth. The effectiveness of a preterm birth prevention service is limited by the ability to predict preterm birth. Managing the risk of preterm birth will involve optimising current treatments (such as progesterone) and adding new experimental treatments [38]. Our detailed findings provide a reference for studies in other settings and a baseline for the introduction of new predictive and preventative interventions.

## Electronic Supplementary Material

Below is the link to the electronic supplementary material.

Supplementary Material 1

## Data Availability

Data is presented within the manuscript tables, additional data that support the findings of this study are available upon reasonable request from the corresponding author.

## Abbreviations

28d: 28 days
Abrupt: Abruption
APH: Antepartum haemorrhage
BMI: Body mass index
Cerc: Cerclage
Chorio: Chorioamnionitis
CS: Caesarean section
ELCS: Elective caesarean section
EMCS: Emergency caesarean section
GDM: Gestational diabetes mellitus
Gest: Gestation
Instrum: Instrumental birth (forceps or ventouse)
IUD: Intrauterine death
IUGR: Intrauterine growth restriction
LLETZ: Large loop excision of the transformation zone
MSU: Mid-stream urine
NA: Not applicable
NR: Not recorded
OC / ICP: Obstetric cholestasis / intrahepatic cholestasis of pregnancy
PAS: Placenta accrete spectrum
PET: Preeclampsia
Plac.: Placenta
PTB: Preterm birth
PPROM: Preterm prelabour rupture of membranes
Prev: Previous
Prog: Progesterone
PV/PR: Per vagina / per rectum
SD: Standard deviation
SGA: Small for gestational age
Spont: Spontaneous
STI: Sexually transmitted infection
SVD: Spontaneous vaginal delivery
US: Ultrasound
UTI: Urinary tract infection
