# Supplementary material for: Evaluation of a two-tier preterm birth prevention service in a tertiary hospital in the United Kingdom: a retrospective cohort study
Source: BMC Pregnancy Childbirth. 2025 Apr 15;25:452. doi: 10.1186/s12884-025-07538-8 (PMC11998394; doi:10.1186/s12884-025-07538-8)
Supplement: Supplementary file 1 — Supplementary Material 1 [file 12884_2025_7538_MOESM1_ESM.docx]

**Table S1. Birth outcomes from other Preterm Birth Clinics and from this study**

| **Publication** | **Outcomes** |
| --- | --- |
| Bolt 2011 | 18% preterm birth rate (<37 weeks) for women from their preterm birth clinic, N=147 |
| Ivandic 2018 | 50% preterm birth rate (<37 weeks) in women treated with Arabin pessary.  N=129 |
| Karkhanis 2012  (conference abstract only – data from Dawes 2020 a) | 32% preterm birth rate (<37 weeks) from their preterm birth clinic.  N=180 |
| Yulia 2015  (conference abstract only – data from Dawes 2020 a) | 8% preterm birth rate (<37 weeks) from their preterm birth clinic (which also included women we would see in the screening clinic).  N= 415 |
| Kindinger 2013 (conference abstract only – data from Dawes 2020 a) | 8% preterm birth rate (<37 weeks) from their preterm birth clinic.  N= 160 |
| Burul 2014  (conference abstract only – data from Dawes 2020 a) | Median age at birth 35+2 weeks from the PTB clinic.  N=125 |
| Grant 2016 (conference abstract only – data from Dawes 2020 a) | 25% preterm birth rate (<37 weeks) from their preterm birth clinic.  N=146 |
| Turitz 2016 | 36% preterm birth rate (<37 weeks) from their preterm birth clinic.  N = 218 |
| Manuck 2011 | 70 women were seen in the PTB clinic and 153 women with similar risk factors had usual obstetric care. All these women had a history of at least one previous spontaneous PTB at <35/50 (mean gestation 30 weeks).   - PTB clinic group:   - 48.6% PTB < 37/40   - 5.7% PTB < 32/40   - Composite neonatal morbidity: 5.7%   - Neonatal death: 3.2% - Control group:   - 63.4% PTB < 37/40   - 13.7% PTB < 32 / 40   - Composite neonatal morbidity: 16.3%   - Neonatal death: 0.7% |
| Hughes 2017 | - 21.4 % preterm birth rate < 37 weeks for women seen in a PTB clinic. N= 756 - Neonatal outcomes (for births > 20/40). N= 747:   - Livebirth 97.3%   - Stillbirths 1.3%   - Neonatal deaths 1.5% |
| Newnham 2017 | - 32% preterm birth rate < 37 weeks for women seen in a preterm birth clinic (for live births, N= 84). - 8.7% pregnancy loss rate (gestation not specified). (N = 92 for live and stillbirths total) |
| Stricker 2016 | - 44% preterm birth rate < 37 weeks, 28% at less than 34/40 - 17% composite poor neonatal outcomes (perinatal or neonatal death, respiratory distress syndrome more than grade II, bronchopulmonary dysplasia, intraventricular haemorrhage grade III or IV, and necrotizing enterocolitis) - 0.9% perinatal death rate - N = 106 |
| Danti 2014 | - 15% preterm birth rate <37 weeks. N= 86 |
| Jin 2021 | - 21.2% preterm birth rate <37 weeks (from PTB clinic) N=294 |
| Dawes 2020 (b) | - 19.5% preterm birth rate < 37 weeks for women seen in PTB clinic, N=309 - 1.6% second trimester loss, 4.6% perinatal death rate |
| Shea 2025 (this study) | - Screening clinic, N = 181   - 0% stillbirth rate   - 3.1% preterm birth rate < 34/40 - Preterm Birth Clinic, N = 79   - 2.7% stillbirth rate   - 16.2% preterm birth rate < 34/40 |

**Table S2: Cases seen in the Preterm Birth Clinic with birth at less than 34 weeks**

| **Gestation** | **Short cervix on any scan** | **Cerclage** | **Progesterone** | **Outcome** |
| --- | --- | --- | --- | --- |
| 23+4 | Yes | Yes | Yes | EMCS at 23+4 weeks following SROM and signs of chorioamnionitis, had abdominal cerclage in situ |
| 25+5 | Yes | Yes | Yes | Spontaneous PTB at 25+5 (SROM at 25+1, suture removed) |
| 29+1 | No | No | Yes | Preterm labour, had EMCS at 29+1 weeks for chorioamnionitis and previous caesarean section |
| 29+3 | No | No | No | EMCS at 29+3 for severe preeclampsia on a background of uncontrolled Type I diabetes and fetal growth restriction |
| 30+0 | No | No | Yes | Spontaneous preterm birth |
| 30+5 | No | Yes | Yes | Abruption, suture removed, spontaneous preterm birth |
| 31+1 | No | No | Yes | Spontaneous preterm birth |
| 32+6 | No | No | Yes | Caesarean section for abruption and breech presentation |
| 33+4 | Yes | Yes | Yes | PPROM, induction for chorioamnionitis, then caesarean section for failure to progress. |
| 33+6 | No | No | No | PPROM with placenta accreta, had caesarean section |
| 33+6 | Yes | No | No | Caesarean section for abruption and previous caesarean section |

**Table S3: Eligibility for history-indicated cerclage and number of cerclages placed**

| **Criteria for history indicated cerclage** | **Number eligible** | **Number had a cerclage** | **Proportion of those eligible who had a cerclage** |
| --- | --- | --- | --- |
| Three or more previous preterm births or second-trimester miscarriages (RCOG criteria) | 7 | 2 | 29% |
| At least one preterm birth at less than 32 weeks  (criteria reportedly used in some PTB clinics) | 21 | 5 | 24% |
| At least one preterm birth at less than 32 weeks or midtrimester miscarriage  (criteria reportedly used in some PTB clinics) | 37 | 14 | 38% |
| “History” listed as the indication for cerclage on the operation note for cases from our Preterm Birth Clinic | 7 out of 26 cerclages placed | | 26.9% |
